# Porcine reproductive and respiratory syndrome virus NSP5 exploited UBE2L6 to promote viral replication via antagonising host RLRs and ISGylation

**DOI:** 10.1186/s13567-025-01558-0

**Published:** 2025-07-03

**Authors:** Zhenbang Zhu, Lulu Chen, Meng Zhang, Qianwen Lin, Yifan Yan, Wenqiang Wang, Wei Wen, Zhendong Zhang, Xiangdong Li

**Affiliations:** 1 Jiangsu Co-Innovation Center for Prevention and Control of Important Animal Infectious Diseases and Zoonoses, College of Veterinary Medicine, Yangzhou University, Yangzhou, 225009 China; 2 Joint International Research Laboratory of Agriculture and Agri-Product Safety, The Ministry of Education of China, Yangzhou University, Yangzhou, 225009 China

**Keywords:** PRRSV, NSP5, UBE2L6, ISGylation, innate immune response

## Abstract

Porcine reproductive and respiratory syndrome virus (PRRSV) inhibits the host innate immune response to promote its replication. The ubiquitin–proteasome system (UPS) and ISGylation both play roles in modulating host innate immunity. Within this process, ISG15-conjugating enzyme E2L6 (UBE2L6) functions as an E2 ubiquitin/ISG15-conjugating enzyme, which is crucial for the enzymatic cascades of UPS and ISGylation. However, the role of UBE2L6 during PRRSV infection remains unclear. Here, we report that UBE2L6 was up-regulated at both the transcript and protein levels during PRRSV infection. Overexpression of UBE2L6 facilitated PRRSV replication, whereas knockdown of UBE2L6 reduced viral replication. Mechanistically, UBE2L6 promoted the degradation of RIG-I and MDA5 protein expression via the ubiquitin–proteasome pathway and decreased ISGylation levels during PRRSV infection, thereby inhibiting the expression of type I interferons and interferon-stimulated genes (ISGs). In addition, UBE2L6 interacted with PRRSV NSP5 and stabilised the NSP5 protein. Together, PRRSV NSP5 and UBE2L6 further facilitated the degradation of RIG-I and MDA5 via the K48-linked ubiquitination pathway, ultimately facilitating PRRSV replication. Notably, UBE2L6 had minimal impact on RIG-I and MDA5 expression in the absence of PRRSV infection. In summary, UBE2L6 regulated host innate immunity and viral replication through its ubiquitination and ubiquitination-like functions. These findings provide novel insights into how PRRSV NSP5 exploits the host UPS to inhibit the innate immune response and deepen our understanding of the mechanism of host-virus interaction.

## Introduction

Porcine reproductive and respiratory syndrome virus (PRRSV) is one of the most economically significant pathogens affecting the global swine industry [1]. PRRSV is an enveloped, positive-stranded RNA virus with a genome size of approximately 15 kb, containing at least 10 open reading frames (ORFs), which are transcribed and translated into viral non-structural proteins (NSP) and structural proteins [2]. The non-structural proteins are involved in viral replication, transcription, and modulation of host cell functions, while the structural proteins are essential for forming the viral particles [3]. The primary target cells for PRRSV infection are porcine alveolar macrophages (PAMs), which are critical components of the innate immune system [4]. This system provides the first line of defence against invading pathogens and plays a key role in activating and modulating the subsequent adaptive immune response. Retinoic acid-inducible gene-I (RIG-I)-like receptors (RLRs), including RIG-I and MDA5, are important pattern recognition receptors (PRRs) that recognise viral RNA in the cytoplasm. This recognition triggers a signalling cascade that activates transcription factors and leads to the production of cytokines and chemokines [5].

However, PRRSV has developed several strategies to evade the host innate immune response by disrupting RLRs. PRRSV degrades MDA5 via autophagy to antagonise innate immunity [6]. For example, the PRRSV nucleocapsid protein can interfere with TRIM25-mediated RIG-I ubiquitination [7]. NSP11 further suppresses mitochondrial antiviral-signalling protein (MAVS) and RIG-I expression, thereby inhibiting type I interferon production [8], while NSP2 degrades SH3KBP1 and lowers K63-linked polyubiquitination of RIG-I, thus inhibiting RIG-I signalling transduction [9]. In addition, NSP4 cleaves virus-induced signalling adaptor (VISA) and other mitochondrial antiviral signalling complexes, suppressing IFN-β expression [10]. Although many viral proteins of PRRSV have been studied for their role in inhibiting the host innate immune response through various mechanisms, the function of PRRSV NSP5, particularly its role in antagonising innate immunity, remains unclear. It was reported that PRRSV NSP5 degrades RLRs through autophagy-related proteins [11], which suggests that NSP5 can inhibit innate immunity. The potential mechanisms by which PRRSV NSP5 inhibits innate immunity require further investigation.

Interferon-stimulated gene 15 (ISG15) is recognised as a type I interferon (IFN)-stimulated reaction protein that can defend against viral invasion and replication [12]. ISG15 also functions as a ubiquitin-like protein that binds to substrate lysine residues through the carboxyl-terminated LRLRGG motif, resulting in ISG15 conjugation, commonly known as ISGylation [13], which plays a crucial role in type I interferon-mediated innate immune responses and demonstrates strong antiviral effects [14]. Meanwhile, the ISGylation of viral proteins, including the influenza A virus (IAV) NS1, infectious bronchitis virus (IBV) NP, and the coxsackievirus B3 virus (CVB3) 2A^Pro, can disrupt the function of these viral proteins and their interaction with host proteins, leading to a suppression of viral replication [15–17]. ISGylation is identified as an essential protein modification that helps protect against viral infections.

Similar to the ubiquitin–proteasome system (UPS), the ubiquitin-activating enzyme E1 (UBE1L), the ubiquitin-binding enzyme E2 (UBE2L6), and the ubiquitin ligase E3 (HERC5, TRIM25, and ARIH1) are essential for the catalysis of ISG substrates [18]. The ISG15-conjugating enzyme E2L6 (UBE2L6) is an E2 ubiquitin/ISG15-conjugating enzyme with active-site cysteines responsible for ubiquitination and ISGylation [19]. Furthermore, UBE2L6 is involved in various pathophysiological processes, including cell proliferation, tumour growth, and the transduction of DNA damage signals [20, 21]. Despite limited research on the relationship between *UBE2L6* and pathogens, it has been identified as one of the core genes consistently up-regulated during SARS-CoV-2 infection, based on integrative transcriptional analysis in vitro and in vivo.

Furthermore, SARS-CoV-2 infection disrupts UBE2L6-mediated ISGylation and promotes the polarisation of M1 macrophages [22]. Seneca virus A (SVA) up-regulates the expression of UBE2L6, which interacts with and facilitates K48 and K63 ubiquitination chains in the three-dimensional (3D) protein of SVA. This process enhances the stability of SVA 3D and promotes viral replication [23]. UBE2L6 is also a bifunctional conjugating enzyme that plays an important role in the level of host ubiquitination and ISGylation. Therefore, we speculated that UBE2L6 has the potential to regulate host innate immune and viral replication via its function as a ubiquitin-conjugating enzyme during PRRSV infection.

In this study, we demonstrated the role of UBE2L6 in regulating the host innate immune response and viral replication during PRRSV infection. We also found that PRRSV NSP5 was involved in the UBE2L6-mediated immune response. For instance, PRRSV infection led to an increase in the expression of UBE2L6, which, in turn, stabilised the PRRSV NSP5 protein. Moreover, PRRSV NSP5 interacted with UBE2L6 to promote the degradation of RIG-I and MDA5 via the UBE2L6 catalytic K48-linked ubiquitination pathway. This interaction inhibited the expression of type I interferons and ISGs, ultimately facilitating PRRSV replication. Briefly, we determined a new mechanism by which PRRSV exploits the UBE2L6-mediated ubiquitin–proteasome pathway to inhibit the innate immune response. This finding provides valuable insights into the interaction between PRRSV and its host, as well as a foundation for preventing and controlling PRRSV.

## Materials and methods

### Cells and viruses

Marc-145 cells, derived from African green monkey kidney cells and permissive for PRRSV infection, were cultured in Dulbecco’s Modified Eagle Medium (DMEM) (Hyclone, USA) supplemented with 10% foetal bovine serum (FBS) (Gibco, USA) and 1% penicillin–streptomycin. Cells were incubated at 37 °C in 5% CO_2_ and passaged every 2–3 days using 0.25% trypsin–EDTA when they reached 80–90% confluency. HEK293T cells, primarily used for plasmid transfection, and PK-15 cells were also maintained in DMEM containing 10% FBS at 37 °C in 5% CO_2_. PAMs were primarily isolated from the bronchoalveolar lavage fluid of 6-month-old piglets using phosphate-buffered saline (PBS) and cultured in RPMI 1640 medium supplemented with 10% FBS and 1% penicillin–streptomycin under identical conditions. Three PRRSV-2 strains were used: CHR6, Li11 (lab-maintained), and XJ17-5 (provided by Professor Nanhua Chen, Yangzhou University, China). All strains were propagated in Marc-145 cells and titrated as the 50% tissue culture infective dose (TCID_50_).

### Expression vector construction and transfection

The *UBE2L6* genes were obtained from PAMs cDNA and subcloned into a pcDNA3.1-myc vector (EK-Bioscience, China), which carries a Myc-tag. The cDNAs encoding *RIG-I, MDA5,* and *UBE2L6* were obtained from PAM cDNA and subcloned into the pcDNA3.1-GFP vector (EK-Bioscience, China) with a GFP tag. Porcine *MDA5* cDNA was cloned into the pcDNA3.1-Flag vector (EK-Bioscience) with a Flag tag. The PRRSV *NSP2–NSP12* genes were amplified from the PRRSV CHR6 strain and cloned into the vector pmCherry-N (632523, Takara, Japan) with an N-terminal mCherry tag. The *ISG15* genes were obtained from PAMs cDNA and subcloned into the pcDNA3.1-FLAG vector containing a Flag tag. An ISG15 mutant was an amino acid substitution in the C-terminal LRLRGG motif (mutated from LRLRGG to LRLRAA), which inhibits ISGylation [24]. The *ISG15* mutant genes were also cloned into the pcDNA3.1-FLAG vector. Ub-HA plasmid and the Ub-48K and Ub-63K mutant plasmids (Ub-K48-HA and Ub-K63-HA) were kindly provided by Professor Yaosheng Chen (Sun Yat-sen University).

Marc-145 and HEK293T cells were seeded in six-well plates and grown to 60–70% confluency. Plasmid transfections were performed using Lipofectamine 2000 (Invitrogen), Lipofectamine 3000 (Invitrogen), or jetPRIME^®^, according to the manufacturer’s protocol. Cells were harvested after 24–36 h for different analyses.

### RNA interference

Small interference RNAs (siRNAs) targeting *UBE2L6* and siRNA-negative control (siNC) were designed and synthesised by GenePharma (Suzhou, China). PAMs were seeded in six-well plates at 2 × 10^6^ cells/well and transfected with the indicated siRNAs at a final concentration of 10 nM using Lipofectamine RNAiMAX (Invitrogen, USA), according to the manufacturer’s instructions. Transfection was carried out for 24 h before further treatment. The siRNA sequences are listed in Table 1.

**Table 1:** **Sequences of siRNAs in this study.**

siRNA	siRNA sequence (5′ − 3′)	
	Sense	Antisense
siUBE2L6-1	GAAGCUGUUCAGCGAUGAUTT	AUCAUCGCUGAACAGCUUCTT
siUBE2L6-2	GCCUGAGAAACCGCCCUACTT	GUAGGGCGGUUUCUCAGGCTT
siUBE2L6-3	GCUGGUGAAUAGACCAGAGTT	CUCUGGUCUAUUCACCAGCTT
siNC	UUCUCCGAACGUGUCACGUTT	ACGUGACACGUUCGGAGAATT

### Quantitative real-time reverse-transcription polymerase chain reaction

Quantitative real-time reverse-transcription polymerase chain reaction (qRT-PCR) was used to measure the mRNA expression of *PRRSV N, UBE2L6, IFN-β, IFN-α, ISG15*, and *ISG56*. Total RNA was extracted from cells using TRIzol reagent (TIANGEN, China). For cDNA synthesis, 1 μg of total RNA was reverse-transcribed using HiScript III RT SuperMix (Vazyme, China) following the manufacturer’s protocol. The qRT-PCR was performed on an Applied Biosystems QuantStudio 3 (Thermo Fisher, USA) using ChamQ Universal SYBR qPCR Master Mix (Vazyme, China). Each 10 µL reaction mixture included: 5 μL of 2 × SYBR Green master mix, 0.4 μL of each forward and reverse primer, 2 μL of cDNA template, and nuclease-free water. Primers were designed to amplify specific target genes and normalised to reference genes *GAPDH* or *HPRT1* (see Table 2 for sequences). All reactions were carried out in triplicate. The relative expression levels of the target genes were calculated using the 2^−ΔΔCt^ method.

**Table 2:** **Primers used for RT-qPCR.**

Primer	Sequence (5′–3′)
*PRRSV N*-F	AAAACCAGTCCAGAGGCAAG
*PRRSV N*-R	CGGATCAGACGCACAGTATG
p*UBE2L6*-F	TTCCCGGAGGAGTACCCATT
p*UBE2L6*-R	AGGGCTTCCAGTTCTCGTTG
p*IFN-α*-F	TCCAGCTCTTCAGCACAGAG
p*IFN-α*-R	AGCTGCTGATCCAGTCCAGT
p*IFN-β*-F	AGCACTGGCTGGAATGAAACCG
p*IFN-β*-R	CTCCAGGTCATCCATCTGCCCA
p*ISG15*-F	ACGGCCATGGGTAGGGA
p*ISG15*-R	TGCTGCTTCAGGTCCGATG
p*ISG56*-F	TCAGAGGTGAGAAGGCTGGT
p*ISG56*-R	GCTTCCTGCAAGTGTCCTTC
p*HPRT1*-F	TGGAAAGAATGTCTTGATTGTTGAAG
p*HPRT1*-R	ATCTTTGGATTATGCTGCTTGACC
m*IFN-α*-F	GGAGGAGTTTGGCAACCAGT
m*IFN-α*-R	TCCCAAGCAGCAGATGAGTC
m*IFN-β*-F	GCAATTGAATGGAAGGCTTGA
m*IFN-β*-R	CAGCGTCCTCCTTCTGGAACT
m*ISG15*-F	CACCGTGTTCATGAATCTGC
m*ISG15*-R	CTTTATTTCCGGCCCTTGAT
m*ISG56*-F	CCTCCTTGGGTTCGTCTACA
m*ISG56*-R	GGCTGATATCTGGGTGCCTA
m*GAPDH*-F	TGACAACAGCCTCAAGATCG
m*GAPDH*-R	GTCTTCTGGGTGGCAGTGAT

^a^F: forward primer, R: reverse primer. The letter “m” indicates that it is for a green monkey gene. The letter “p” indicates that it is for a pig gene.

^b^Pig gene sequences, green monkey gene sequences, and PRRSV gene sequences were downloaded from GenBank.

### Detection of gene expression of type I interferon and interferon-stimulated genes

Marc-145 cells were mock-transfected or transfected with *UBE2L6* plasmids for 24 h, then infected with PRRSV at a multiplicity of infection (MOI) of 1 for 0, 12, 24, and 36 h. In a separate experiment, Marc-145 cells were transfected with varying concentrations of *UBE2L6* plasmids and infected with PRRSV (MOI = 1) for 36 h. Additionally, Marc-145 cells were transfected with *UBE2L6* interference RNA and infected with PRRSV (MOI = 1) for 3, 6, and 12 h. Relative expression of *IFN-β, IFN-α, ISG15,* and *ISG56* was measured using qRT-PCR as described above. Each treatment group included three biological replicates.

### Western blot

Cells were mock-treated, treated with corresponding reagents or infected with PRRSV at the indicated time points. After harvesting, cells were washed with PBS and lysed in RIPA buffer (Beyotime, China) supplemented with protease and phosphatase inhibitor cocktails. Lysis was performed on ice for at least 30 min. Supernatants were collected after centrifugation at 12 000 × *g* for 15 min at 4 °C. Protein concentration was quantified, and equal amounts of protein were subjected to 8–12% sodium dodecyl sulfate–polyacrylamide gel electrophoresis (SDS-PAGE). Following electrophoresis, proteins were transferred onto polyvinyl difluoride (PVDF) membranes (Merck Millipore, USA), which were then blocked with 5% non-fat dry milk or bovine serum albumin (BSA) in TBST (20 mM Tris–HCl, pH8.0, 150 mM NaCl, 0.05% Tween 20) for 1 h at room temperature with gentle shaking. Membranes were incubated overnight at 4 °C with specific primary antibodies diluted in 5% BSA in TBST.

The primary antibodies used in this study included: anti-PRRSV N (4A5) antibody (9041) (MEDIAN, Republic of Korea). ISG15 rabbit polyclonal antibody (15981), UBE2L6 rabbit polyclonal antibody (17278), GAPDH mouse monoclonal antibody (60004), MDA5 rabbit polyclonal antibody (21,775), RIG-I rabbit polyclonal antibody (20566), ubiquitin rabbit polyclonal antibody (10201), HA rabbit polyclonal antibody (51064), and GFP rabbit polyclonal antibody (50430) were all purchased from Proteintech Group (Proteintech, USA). Myc-tag (9B11) mouse mAb (2276) and RIG-I rabbit monoclonal antibody (D14G6) were purchased from Cell Signalling Technology (CST, USA). Flag mouse monoclonal antibody (M20008) was purchased from Abmart (Abmart, China). Anti-mCherry antibody (ab183628) was purchased from Abcam (Abcam, England).

After three washes in TBST, the membranes were incubated for 1 h at room temperature using corresponding secondary antibodies (diluted 1:5000). The blots were visualised using an enhanced chemiluminescence detection kit (ECL; NCM Biotech, China) and a chemiluminescence imaging system (Tanon, Shanghai, China).

### Co-immunoprecipitation

Cells were transfected or co-transfected with indicated plasmids for 24 h or transfected and subsequently infected with PRRSV for the indicated time points. At harvest, cells were lysed on ice in IP lysis buffer (Beyotime, China) supplemented with protease and phosphatase inhibitor cocktails (Beyotime, China) for 30 min. Lysates were centrifuged at 10 000 × *g* for 15 min at 4 °C to collect the supernatants for standby application. Approximately 3 μg of each indicated antibody was conjugated to 30 μL of magnetic beads for 2–3 h at room temperature using the Dynabeads™ Protein G Immunoprecipitation Kit (10007D, Invitrogen, USA).

Subsequently, the obtained supernatants were incubated with magnetic bead-Ab complex overnight at 4 °C with gentle rotation. Beads were washed four times with lysis buffer, and immunoprecipitated complexes were eluted by boiling in SDS sample buffer for 10 min. The precipitates and whole cell lysates were analysed using western blot.

### Immunofluorescence

HEK293T, Marc-145, and PK-15 cells were seeded onto sterile glass coverslips in 12-well culture plates and then transfected with plasmids or infected with PRRSV for the indicated time points. Cells were gently washed three times with PBS and fixed with 4% paraformaldehyde for 10 min at room temperature to preserve cell morphology and antigenicity. The fixed cells were permeabilised using 0.5% Triton X-100 in PBS for 15 min at room temperature. After three further PBS washes, cells were blocked with 3% BSA in PBS for 1 h at room temperature. Following blocking, cells were incubated with primary antibodies overnight at 4 °C. After rinsing with PBS, the cells were incubated with fluorophore-conjugated secondary antibodies for 1 h at room temperature in the dark. Nuclear counterstaining was performed using 4ʹ,6-diamidino-2-phenylindole dihydrochloride (DAPI; Beyotime, China) diluted in PBS.

After counterstaining, the cells were rewashed with PBS, and the coverslips were mounted onto microscope slides. Images were captured using either an inverted fluorescence microscope (U-HGLGPS, OLYMPUS, Japan) or a confocal laser scanning microscope (LSM 880NLO, Carl Zeiss, Germany). Fluorescence intensity and localisation of the target antigens were analysed using ImageJ software.

### Statistical analysis

Statistical analysis was performed using GraphPad Prism 5.0. Data are presented as mean ± standard error of the mean (SEM). Statistical significance was determined using Student’s *t*-test or one-way analysis of variance (ANOVA). A *p*-value of < 0.05 was considered statistically significant.

## Results

### PRRSV infection promoted the expression of UBE2L6 in PAMs

**Figure 1:**
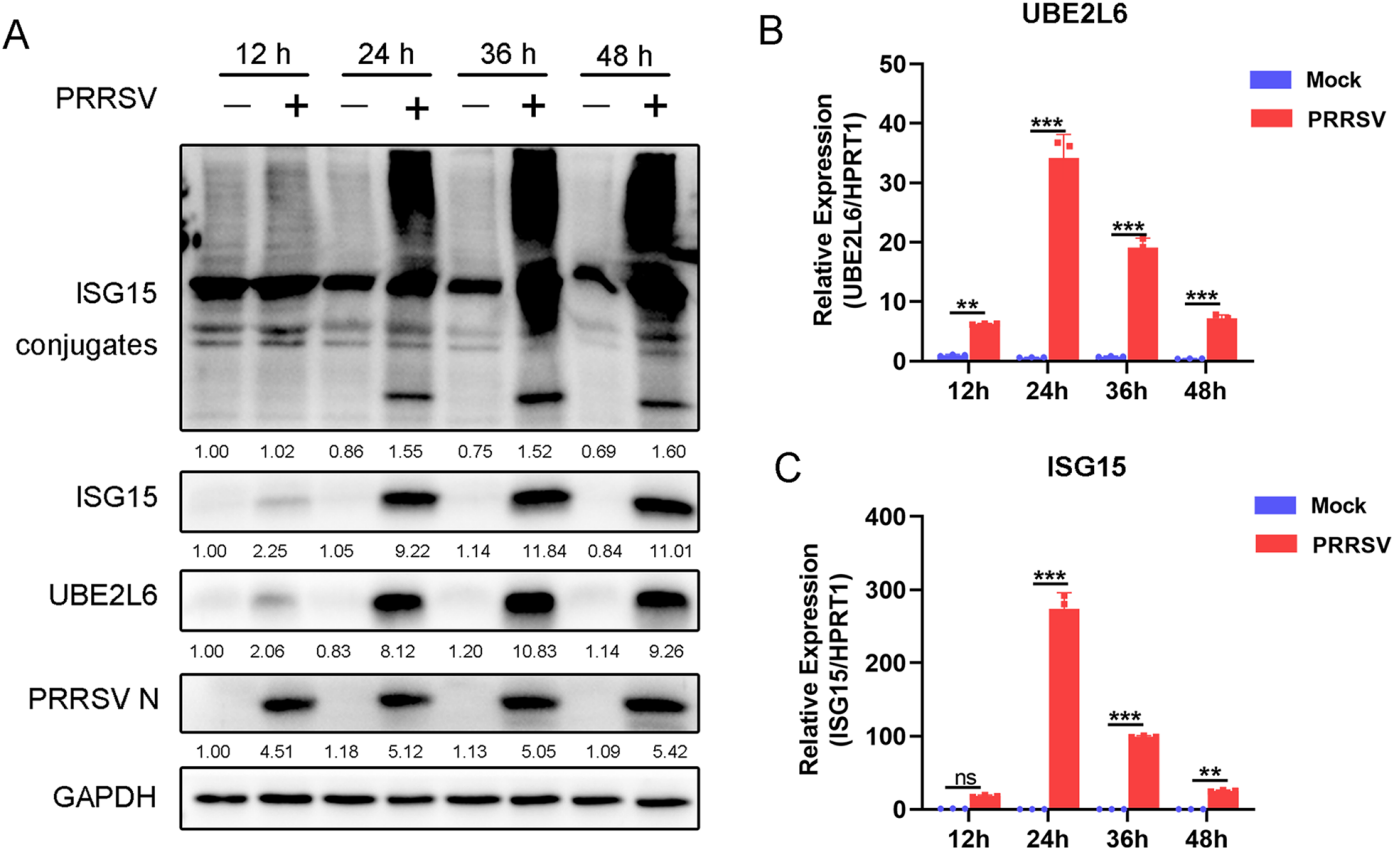
**PRRSV infection promoted the expression of UBE2L6.** **A** PAMs were infected with PRRSV (MOI = 1) for the indicated time (12, 24, 36, and 48 hpi). The protein expression of PRRSV N, UBE2L6, ISG15, and ISG15 conjugates was detected by using western blot. GAPDH is shown as an internal control. For the western blot, the relative band density was normalised to the loading control GAPDH and then compared to the corresponding control. **B**, **C** PAMs were infected with PRRSV (MOI = 1) at 12, 24, 36, and 48 hpi. The transcription levels of *UBE2L6* (**B**) and *ISG15* (**C**) were shown using qRT-PCR. *HPRT1* is shown as an internal control. Data are the results of three independent experiments (means ± SE). Significant differences are denoted by *p < 0.05, **p < 0.01, and ***p < 0.001.

UBE2L6 is a specific enzyme that catalyses ISGylation by conjugating ISG15 to target proteins. To investigate the expression of *UBE2L6*, *ISG15*, and *ISG15* conjugates during PRRSV infection, PAMs were infected with PRRSV and sampled at various time points. As shown in Figure 1A, UBE2L6 protein expression was up-regulated following PRRSV infection, with levels peaking at 36 and 48 h post-infection (hpi). In addition, expression of ISG15 and ISG15 conjugates was also elevated when PAMs were infected with PRRSV.

We further examined the mRNA expression of *UBE2L6* and *ISG15*. *UBE2L6* mRNA expression was significantly increased at 24 hpi and remained higher in PRRSV-infected cells than in mock-infected cells (Figure 1B). The mRNA expression of *ISG15* was also consistent with *UBE2L6* during PRRSV infection (Figure 1C). Taken together, it is likely that PRRSV facilitated *UBE2L6* expression, which suggests it is likely related to PRRSV infection.

### UBE2L6 promoted PRRSV replication

**Figure 2:**
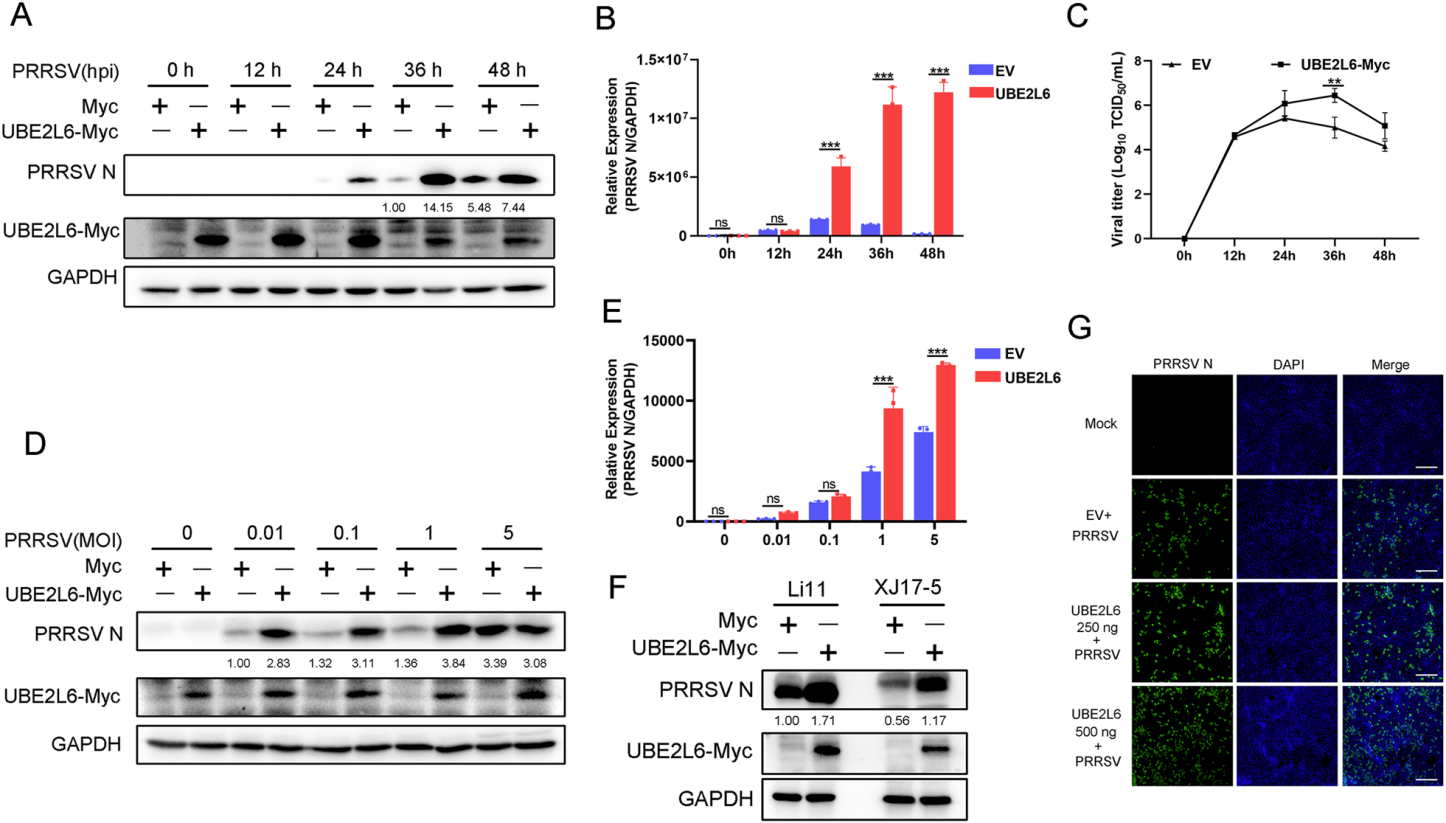
**UBE2L6 overexpression facilitated PRRSV replication.** **A**–**C** Marc-145 cells were mock-transfected or transfected with Myc-tagged *UBE2L6* plasmids for 24 h and then infected with PRRSV (MOI = 1) for indicated time points (0, 12, 24, 36, and 48 hpi). **A** Western blot was used to measure the expression of PRRSV N and UBE2L6-Myc. GAPDH is shown as an internal control. **B** The mRNA expression of PRRSV *ORF7 (N)* was detected by using RT-qPCR. **C** Cell supernatants were collected for the measurement of TCID_50_. **D**–**E** Marc-145 cells were transfected with empty vector or Myc-tagged *UBE2L6* plasmids for 24 h and infected with different MOIs (0, 0.01, 0.1, 1, and 5) of PRRSV. **D** The expression of PRRSV N, Myc, and GAPDH was shown, as measured by western blot analysis; GAPDH served as an internal control. **E** The transcript level of PRRSV *ORF7 (N)* was detected by RT-qPCR. *GAPDH* is shown as an internal control. **F** Marc-145 cells were mock-transfected or transfected with *UBE2L6* plasmids for 24 h, followed by PRRSV Li11 strain and XJ17-5 strain infection for 36 h. Western blot was used to measure the expression of PRRSV N and UBE2L6-Myc. GAPDH is shown as an internal control. **G** Marc-145 cells were transfected with different concentrations of *UBE2L6* plasmids (0, 250, and 500 ng) and then infected with PRRSV (MOI = 1) for 36 h. Immunofluorescence analysis of PRRSV N (green) expression in Marc-145 cells. Nuclei were counterstained with DAPI (blue). Bar = 200 μm. Data are the results of three independent experiments (means ± SE). Significant differences are denoted by *p < 0.05, **p < 0.01, and ***p < 0.001. For the western blot, the relative band density was normalised to the loading control GAPDH and then compared to the corresponding control.

To investigate the role of UBE2L6 affecting PRRSV replication, we constructed a *UBE2L6*-overexpression plasmid. Marc-145 cells were transfected with *UBE2L6* plasmids and subsequently infected with PRRSV for the indicated time points. As shown in Figure 2A, UBE2L6 overexpression facilitated PRRSV replication as indicated by increased levels of the viral nucleocapsid (N) protein at 24, 36, and 48 hpi. In parallel, the level of viral *ORF7* mRNA was significantly elevated in UBE2L6-overexpressed cells at 24, 36, and 48 hpi (Figure 2B). Compared with empty vector transfection, the viral titres were enhanced when cells were transfected with *UBE2L6* plasmids, especially at 36 hpi (Figure 2C). Marc-145 cells were also mock-transfected or transfected with UBE2L6 plasmids and then infected with varying MOIs of PRRSV. UBE2L6 overexpression accelerated PRRSV replication at the MOIs of 0.01, 0.1, and 1, as demonstrated by the increased amount of PRRSV N (Figure 2D). Likewise, mRNA expression of *PRRSV N* was increased at MOIs of 1 and 5 in UBE2L6 overexpressed cells (Figure 2E).

**Figure 3:**
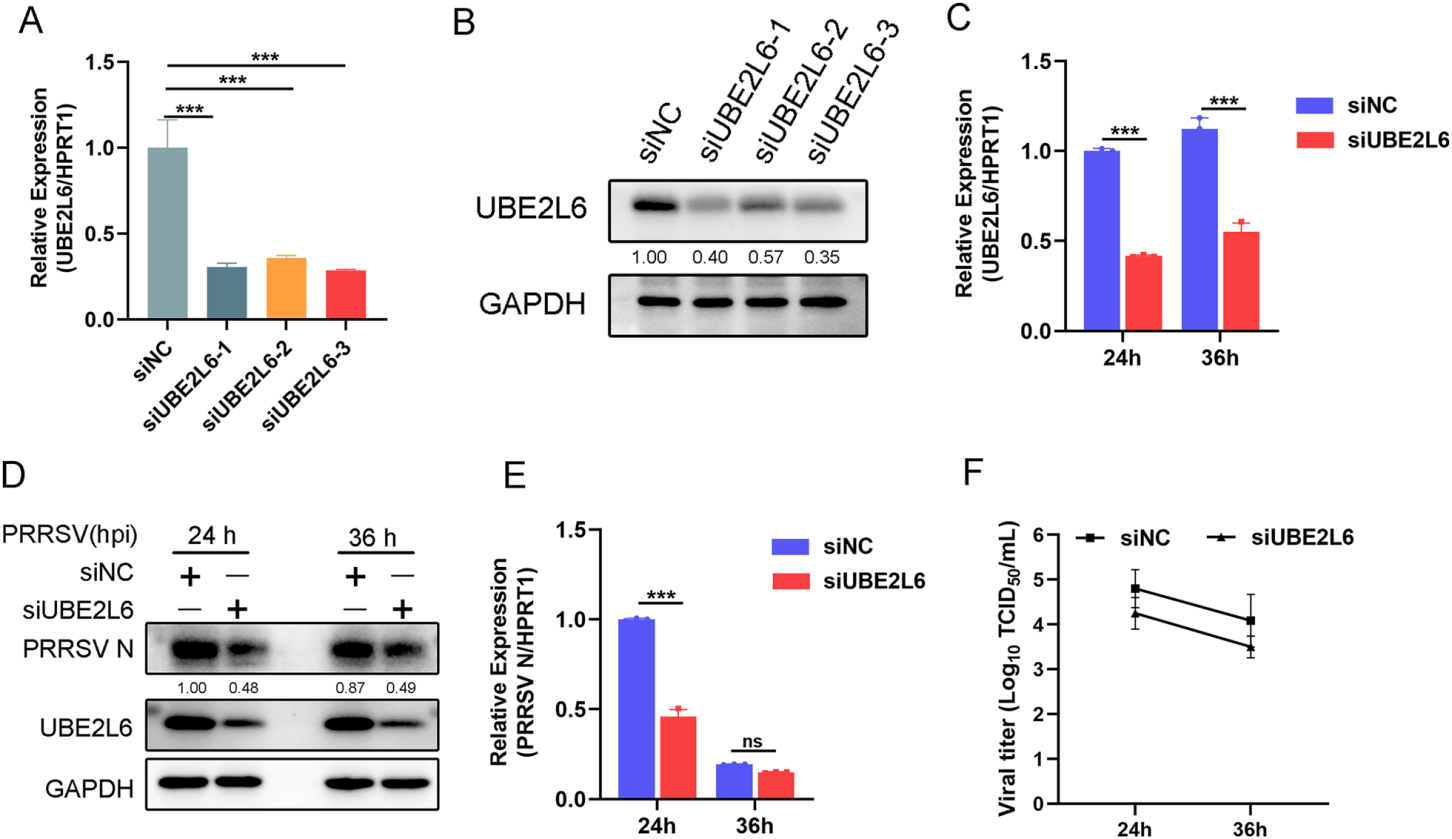
**Silencing of UBE2L6 restrained PRRSV replication.** **A**, **B** PAMs were transfected with scrambled siRNA (siNC) or three *UBE2L6* siRNAs (siUBE2L6) for 24 h, the interfering efficiency of *UBE2L6* was shown using qRT-PCR analysis (**A**) and western blot analysis (**B**). **C**–**F** PAMs were transfected with siNC or siUBE2L6-1 for 24 h and then infected with PRRSV (MOI = 1) for 24 h and 36 h. **C** *UBE2L6* mRNA expression was detected by RT-qPCR. **D** Protein levels of UBE2L6 and PRRSV N were shown by using western blot. **E** PRRSV *ORF7 (N)* transcript level was shown, as detected by RT-qPCR. **F** Viral titers were measured and shown as TCID_50_. Data are the results of three independent experiments (means ± SE). Significant differences are denoted by *p < 0.05, **p < 0.01, and ***p < 0.001. For the western blot, the relative band density was normalised to the loading control GAPDH and then compared to the corresponding control.

To confirm these findings, two additional PRRSV strains (Li11 and XJ17-5) were tested. UBE2L6 overexpression markedly enhanced the replication of both strains, as evidenced by increased expression of UBE2L6-Myc and PRRSV N protein (Figure 2F). Furthermore, varying concentrations of *UBE2L6* plasmids were transfected into Marc-145 cells, and immunofluorescence analysis revealed a significant promotion of viral replication in Marc-145 cells with UBE2L6 overexpression in a dose-dependent manner (Figure 2G). These data suggested that UBE2L6 overexpression promotes PRRSV replication. Conversely, we designed three small interfering RNAs (siRNA) to down-regulate *UBE2L6* expression in PAMs. All three siRNAs showed high interfering efficiency at both the transcriptional and protein levels (Figures 3A, B). Knockdown of UBE2L6 suppressed PRRSV replication, as shown by decreased levels of PRRSV N protein (Figures 3C, D).

Silencing *UBE2L6* also reduced the mRNA level of *PRRSV ORF7* compared with controls (Figure 3E). PRRSV titres were also reduced in *UBE2L6*-silenced cells (Figure 3F). Taken together, these results demonstrate that UBE2L6 overexpression facilitates PRRSV replication, while silencing of *UBE2L6* inhibits it.

### UBE2L6 inhibited the RIG-I signalling pathway and intracellular ISGylation

**Figure 4:**
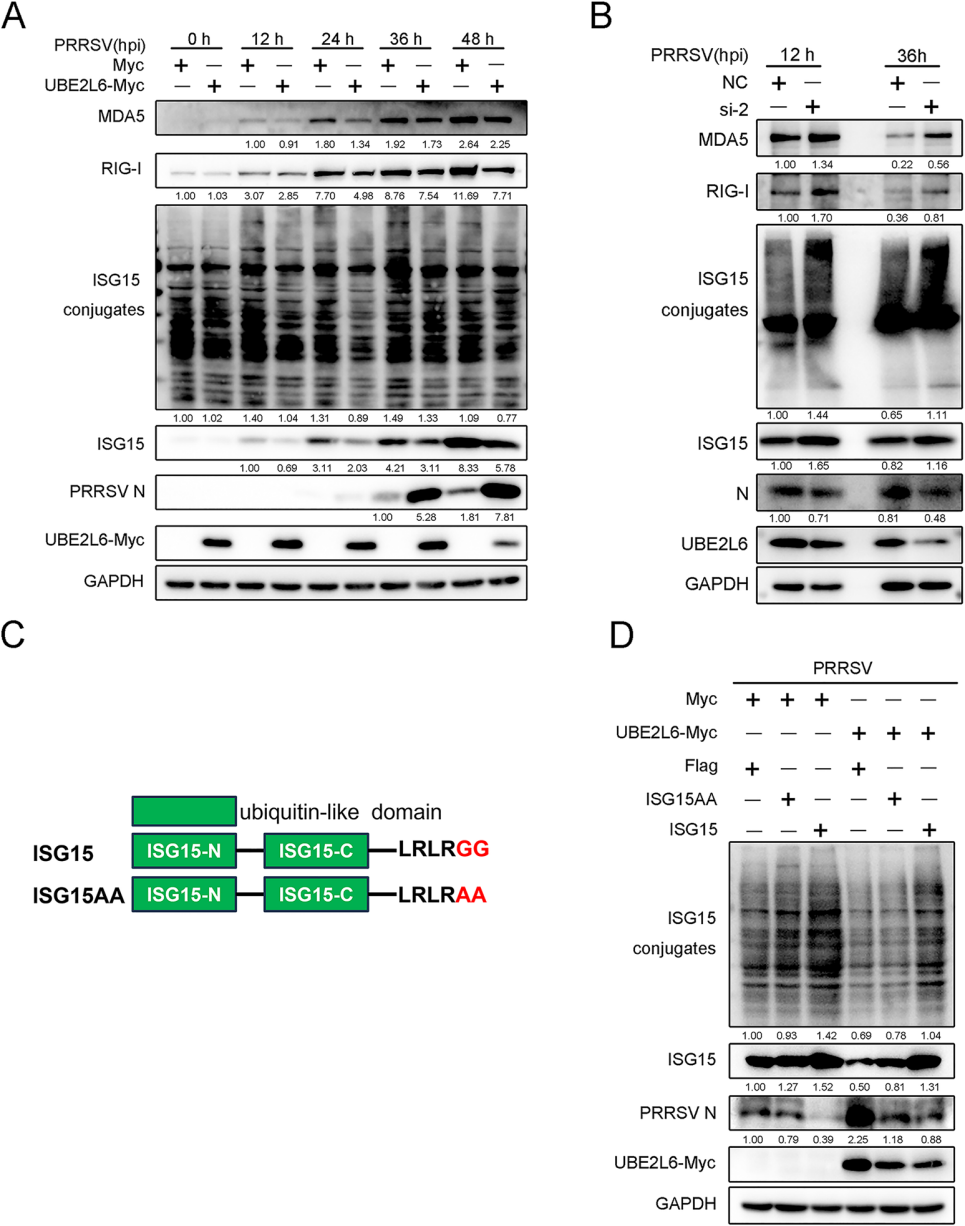
**UBE2L6 inhibited RLRs expression and ISGylation during PRRSV infection.** **A** Marc-145 cells were mock-transfected or transfected with Myc-tagged *UBE2L6* plasmids for 24 h and then infected with PRRSV (MOI = 1) for indicated time points (0, 12, 24, 36, and 48 hpi). Western blot was used to measure the expression of MDA5, RIG-I, ISG15, ISG15 conjugates, PRRSV N, UBE2L6-Myc, and GAPDH. GAPDH is shown as an internal control. **B** PAMs were transfected with siNC or siUBE2L6-1 for 24 h and then infected with PRRSV (MOI = 1) for 12 h and 36 h. Western blot was used to measure the expression of MDA5, RIG-I, ISG15, ISG15 conjugates, PRRSV N, UBE2L6, and GAPDH. GAPDH is shown as an internal control. **C** Schematic representations of ISG15 and mutant ISG15-AA, in which Ala-Ala amino acids were substituted for Gly-Gly in the C-terminal LRLRGG motif. **D** Marc-145 cells were transfected with empty vector or *UBE2L6* plasmid in addition to *ISG15* and *ISG15-AA* plasmids, respectively, and then infected with PRRSV (MOI = 1) for 24 h. The expression of ISG15, ISG15 conjugates, PRRSV N, and GAPDH was measured, as shown by western blot. GAPDH is shown as an internal control.

Adaptor proteins in the RIG-I signalling pathways regulate the host innate immune response through ubiquitination and ISGylation [25]. UBE2L6 is an E2 ubiquitin/ISG15-conjugating enzyme associated with both of these processes. To determine whether UBE2L6 regulates RIG-I/MDA5 protein expression and intracellular ISGylation via its dual enzyme function, Marc-145 cells were mock-transfected or transfected with *UBE2L6* plasmids and then infected with PRRSV at the indicated time points. Overexpression of UBE2L6 facilitated PRRSV replication compared with mock-transfected cells. At that same time, protein expression of RIG-I, MDA5, and ISG15 was noticeably reduced. The level of intracellular ISG15 conjugates was also lower in UBE2L6-overexpressing cells than in control-transfected cells, particularly at 24 hpi (Figure 4A). Conversely, silencing *UBE2L6* clearly enhanced the protein expression of RIG-I, MDA5, and ISG15, along with a marked increase in ISG15 conjugates (Figure 4B). These results suggest that UBE2L6 suppresses the expression of associated proteins in the RIG-I signalling pathways and the overall level of intracellular ISGylation.

To further evaluate the antiviral role of ISGylation during PRRSV infection, we used a conjugation-defective ISG15 mutant containing a Gly-Gly to Ala-Ala substitution in the C-terminal LRLRGG motif sequence (designated ISG15-AA; Figure 4C). Marc-145 cells were transfected with either an empty vector or a *UBE2L6* plasmid, in addition to *ISG15* and *ISG15-AA* plasmids, respectively. After 24 h of transfection, cells were infected with PRRSV for a further 24 h.

As shown in Figure 4D, the level of ISG15 conjugates was significantly reduced in the cells with conjugation-defective ISG15. This was accompanied by increased PRRSV replication compared with normal ISG15-transfected cells. In UBE2L6-overexpressing cells, the integral level of ISG15 conjugates was lower than in empty vector-transfected groups. At the same time, the expression of PRRSV N protein was higher in UBE2L6 overexpressed cells. Moreover, viral replication was also rescued in cells overexpressing UBE2L6 and containing the ISG15 mutant, in contrast to cells with only the ISG15 mutant or those overexpressing both UBE2L6 and ISG15. The findings suggest that ISGylation exerts antiviral effects, which UBE2L6 counteracts via its enzymatic activity. Together, these data indicate that UBE2L6 inhibits the RIG-I signalling pathway and suppresses ISGylation, thereby promoting PRRSV replication.

### UBE2L6 facilitated the degradation of RIG-I/MDA5 through K48-linked ubiquitination

**Figure 5:**
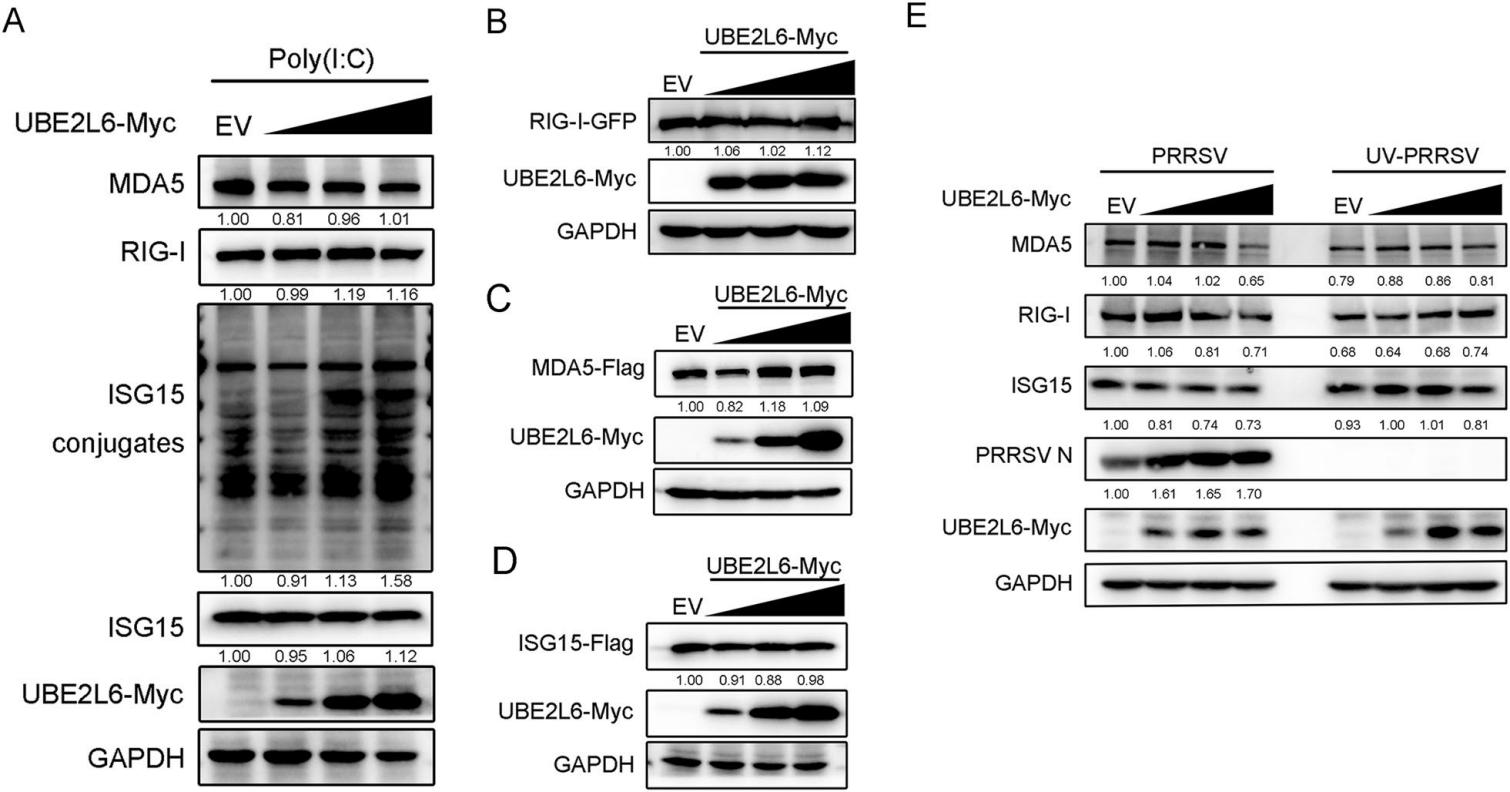
**UBE2L6 had no impact on the expression of RLRs in the absence of PRRSV infection.** **A** Different concentrations of *UBE2L6* plasmids (250, 500, and 1000 ng) were transfected into Marc-145 cells, followed by poly(I:C) stimulation. Western blot was used to measure the expression of MDA5, RIG-I, ISG15, ISG15 conjugates, and GAPDH. GAPDH is shown as an internal control. (B-D) HEK293T cells were co-transfected with different concentrations of *UBE2L6* plasmids (250, 500, and 1000 ng) and GFP-tagged *RIG-I* (**B**), Flag-tagged *MDA5* (**C**), or Flag-tagged *ISG15* (**D**), respectively. Antibodies, including GFP, Flag, Myc, and GAPDH, were used to detect corresponding protein expression. GAPDH is shown as an internal control. **E** Marc-145 cells were transfected with different concentrations of *UBE2L6* plasmids (250, 500, and 1000 ng) and then infected with PRRSV and UV-PRRSV for 36 h. Cells were collected to detect the protein expression of MDA5, RIG-I, ISG15, PRRSV N, and UBE2L6-Myc, which were shown using western blot. GAPDH is shown as an internal control. The relative band density was normalised to the loading control GAPDH and then compared to the corresponding control.

To investigate the mechanism by which UBE2L6 degrades RIG-I, MDA5, and ISG15 protein expression, Marc-145 cells were transfected with varying concentrations of *UBE2L6*-expressing plasmids in the presence of poly(I:C), used to simulate viral nucleic acid stimulation. Surprisingly, except for an increase in ISG15 conjugates, the expression levels of RIG-I, MDA5, and ISG15 remained unchanged across the different concentrations of UBE2L6 under RNA mimic stimulation (Figure 5A). Subsequently, HEK293T cells were co-transfected with varying concentrations of Myc-tagged *UBE2L6* plasmids and plasmids encoding GFP-tagged *RIG-I,* Flag-tagged MDA5, or Flag-tagged *ISG15*. UBE2L6 overexpression did not affect the expression level of RIG-I, MDA5, or ISG15 in these cells, which was quite different from that of PRRSV infection (Figures 5B–D).

To confirm that PRRSV infection is required for UBE2L6-mediated RLRs degradation, Marc-145 cells were transfected with different concentrations of *UBE2L6* plasmids and then infected with either PRRSV or UV-inactivated PRRSV. As expected, the expression of RIG-I, MDA5, and ISG15 was visibly reduced at the highest concentration (1000 ng) of UBE2L6 in PRRSV-infected cells, with only slight decreases observed at 250 and 500 ng. In contrast, no notable changes were detected in the expression level of RIG-I, MDA5, and ISG15 in UV-PRRSV-infected cells, even when UBE2L6 was overexpressed (Figure 5E). These results suggest that PRRSV infection is responsible for UBE2L6-mediated degradation of RIG-I/MDA5 proteins.

**Figure 6:**
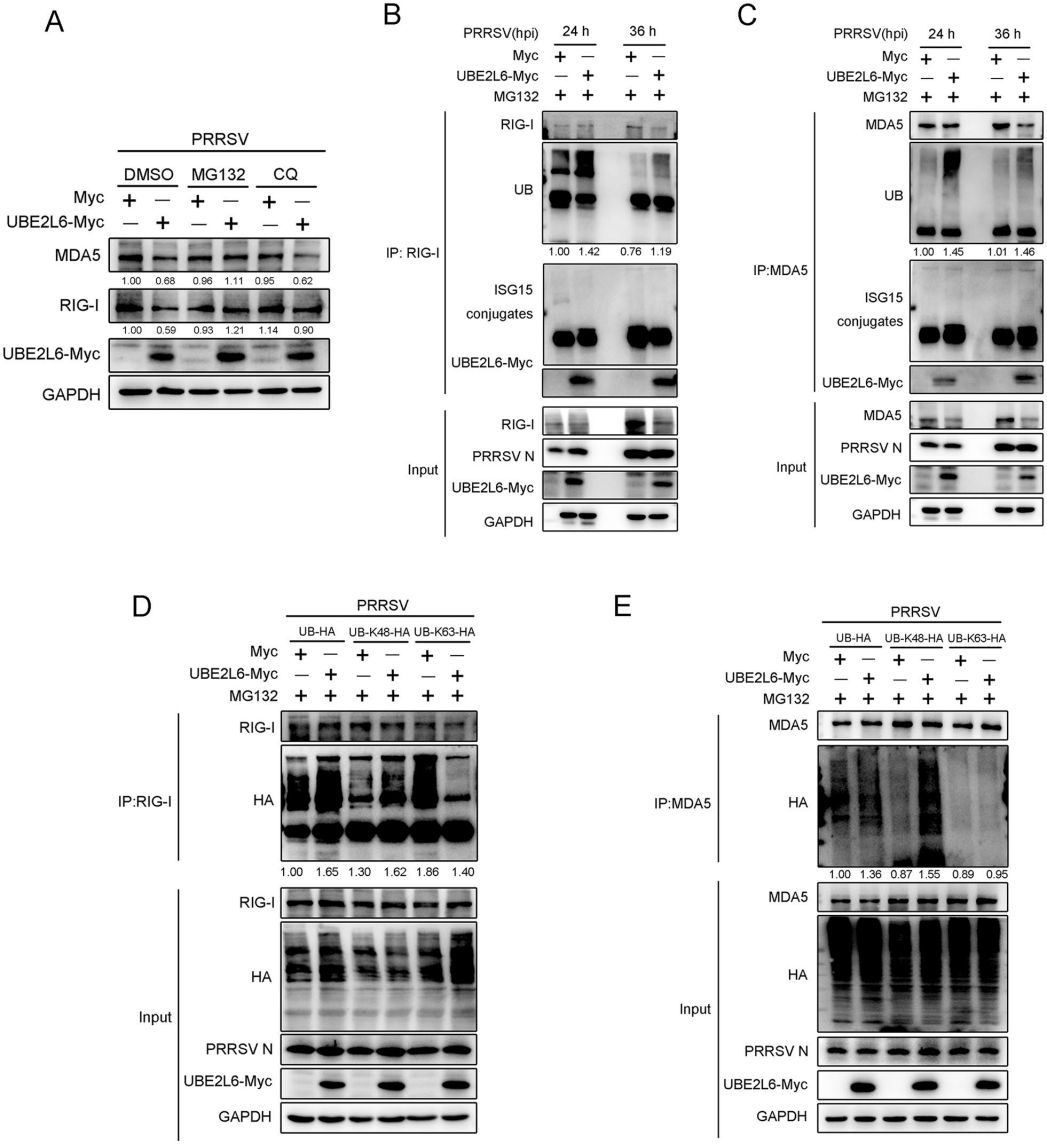
**UBE2L6 inhibited RIG-I/MDA5 expression via K48-linked ubiquitination pathway.** **A** Plasmids expressing UBE2L6 were transfected into Marc-145 cells for 24 h. After transfection, cells were infected with PRRSV (MOI = 1) for 12 h, followed by treatment with DMSO, MG132 (10 μM), and chloroquine (CQ, 50 μM) for another 24 h. Western blot was used to measure the expression change of RIG-I, MDA5, and UBE2L6-Myc. GAPDH was shown as an internal control. **B**, **C** Marc-145 cells were mock-transfected or transfected with Myc-tagged *UBE2L6* plasmids. Cells were then infected with PRRSV (MOI = 1) for 24 hpi and 36 hpi. Before cell collection, cells were treated with MG132 (10 μM) for another 6 h. Cell lysates were immunoprecipitated with RIG-I (**B**) and MDA5 (**C**) antibodies, respectively. The immunoblots of Ub, ISG15 conjugates, UBE2L6-Myc, and RIG-I were shown. **D**, **E** Marc-145 cells were co-transfected with the plasmids expressing Myc-tagged *UBE2L6* and Ub-HA, Ub-K48-HA, or Ub-K63-HA plasmids for 24 h, respectively. Subsequently, cells were infected with PRRSV (MOI = 1) for 36 h and treated with MG132 (10 μM) for another 6 h. Co-IP was used to detect the ubiquitination level of RIG-I (**D**) and MDA5 (**E**) using corresponding antibodies. Immunoprecipitates were analysed by immunoblotting with HA, RIG-I, UBE2L6-Myc, and GAPDH antibodies. GAPDH was shown as an internal control. For the western blot, the relative band density was normalised to the loading control GAPDH and then compared to the corresponding control.

To identify the degradation pathway governed by UBE2L6 during PRRSV infection, the ubiquitin–proteasome inhibitor MG-132 and lysosome inhibitor chloroquine (CQ) were used. As shown in Figure 6A, UBE2L6 overexpression inhibited the expression of MDA5 and RIG-I in dimethyl sulfoxide (DMSO) treated PRRSV-infected cells. Compared with the DMSO control group, the expression of MDA5 and RIG-I increased in overexpressed UBE2L6 cells with MG132 treatment, comparable to the bands in mock-transfected cells with MG132 treatment. However, RIG-I and MDA5 expression remained suppressed or slightly increased upon CQ treatment. These data illustrate that overexpression of UBE2L6 inhibits RIG-I and MDA5 expression via the ubiquitin–proteasome pathway following PRRSV infection.

Furthermore, Marc-145 cells were transfected with the *UBE2L6*-expressing plasmid and then infected with PRRSV for 24 h and 36 h. Before cell collection, MG-132 was added to the cells for another 6 h. A co-immunoprecipitation (co-IP) assay was performed using anti-RIG-I and anti-MDA5 antibodies, followed by immunoblotting with anti-Ub and anti-ISG15 antibodies, respectively. As shown in Figure 6B, endogenous RIG-I interacted with UBE2L6, and its ubiquitination level was enhanced at both 24 hpi and 36 hpi. Similarly, co-IP assays revealed that UBE2L6 also immunoprecipitated with MDA5, and the Ub conjugate level in MDA5 was visibly increased in UBE2L6 overexpressed cells during PRRSV infection (Figure 6C). However, the ISGylation levels of RIG-I and MDA5 remained unchanged (Figures 6B, C).

To further clarify the type of ubiquitinated mechanism involved in UBE2L6 degrading RIG-I/MDA5, Ub-HA, Ub-48K-HA, and Ub-63K-HA plasmids, indicating K48-linked or K63-linked, were co-transfected with *UBE2L6* plasmid. IP assays showed that UBE2L6 overexpression up-regulated the ubiquitination level of endogenous RIG-I. Simultaneously, compared to the control group, K48-linked polyubiquitination of RIG-I was also enhanced in cells overexpressing UBE2L6. In addition, K63-linked polyubiquitination of RIG-I was drastically reduced (Figure 6D). A similar analysis, using IP assays, was performed to detect how UBE2L6 reduces MDA5 expression during PRRSV infection. As shown in Figure 6E, UBE2L6 promoted the ubiquitination and K48-linked ubiquitination level of MDA5 when compared with the control group. There was no observable change in the K63-linked ubiquitination level of MDA5, even in the cells overexpressing UBE2L6. Taken together, these results demonstrate that UBE2L6 facilitates K48-linked ubiquitination of RIG-I and MDA5 during PRRSV infection, resulting in the degradation of RIG-I and MDA5.

### UBE2L6 suppressed the expression of type I interferon and interferon-stimulated genes

**Figure 7:**
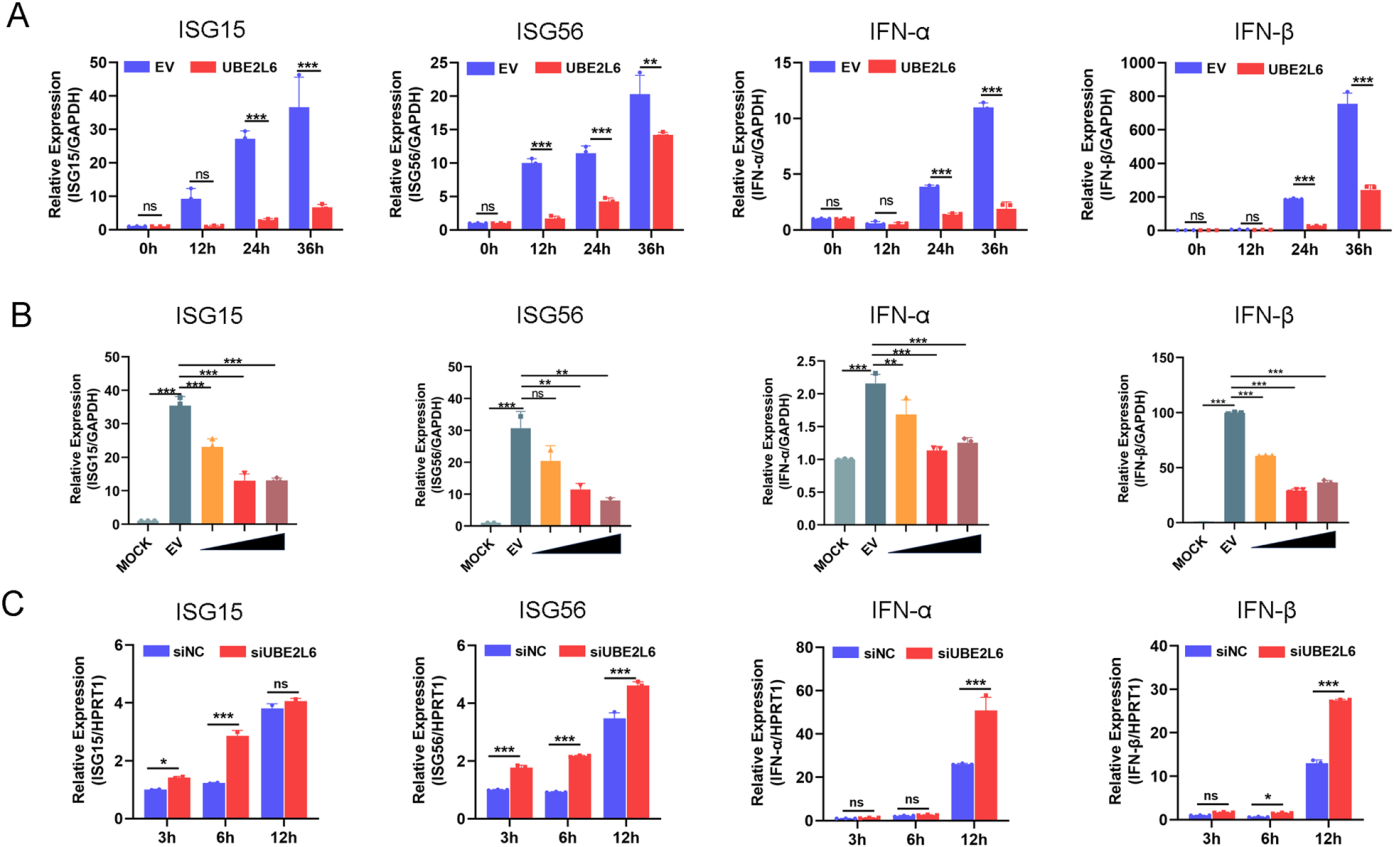
**UBE2L6 suppressed the expression of ISGs and type I interferon during PRRSV infection.** **A** Marc-145 cells were transfected with control plasmids and *UBE2L6* plasmid for 24 h, and then cells were infected with PRRSV (MOI = 1) at the indicated time (0, 12, 24, and 36 hpi). The relative expression of *ISG15*, *ISG56*, *IFN-α*, and *IFN-β* was detected using qRT-PCR, and *GAPDH* served as an internal control. **B** Marc-145 cells were mock-transfected or transfected with different concentrations of *UBE2L6* plasmids for 24 h and then infected with PRRSV (MOI = 1) for another 36 h. The relative expression of *ISG15*, *ISG56*, *IFN-α*, and *IFN-β* was detected using qRT-PCR, and *GAPDH* served as an internal control. **C** PAMs were transfected with siNC and siUBE2L6 for 24 h and additionally infected with PRRSV (MOI = 1) for the indicated time points (3, 6, and 12 hpi). The relative expression of *ISG15*, *ISG56*, *IFN-α*, and *IFN-β* was detected using qRT-PCR. Data are the results of three independent experiments (means ± SE) and normalised to *GAPDH* in each sample. Significant differences are denoted by *p < 0.05, **p < 0.01, and ***p < 0.001.

UBE2L6 plays a role in antagonising the innate immune response by promoting the degradation of RIG-I and MDA5 during PRRSV infection. Briefly, we aimed to determine whether UBE2L6 also affects downstream cytokine expression in the presence of UBE2L6 overexpression. Marc-145 cells were mock-transfected or transfected with *UBE2L6*-expressing plasmids for 24 h, then infected with PRRSV at the indicated time points. Compared with mock-transfected controls, mRNA levels of *ISG15* and *ISG56* were significantly decreased in UBE2L6-overexpressing cells, particularly at 12, 24, and 36 hpi (Figure 7A). The transcript level of *IFN-α* and *IFN-β* was also markedly reduced at 24 and 36 hpi in cells overexpressing UBE2L6 (Figure 7A).

In addition, Marc-145 cells were mock-transfected or transfected with different concentrations of *UBE2L6* plasmids for 24 h, then infected with PRRSV (MOI = 1) for another 36 h. Compared with the mock-infected group, cells transfected with the empty vector exhibited increased expression of *ISG15, ISG56, IFN-α*, and *IFN*-β at 36 hpi. However, UBE2L6 overexpression down-regulated mRNA expression of these genes in a dose-dependent manner relative to the empty vector group (Figure 7B). Furthermore, RNA interference was used to silence *UBE2L6* expression in PAMs, which were then infected with PRRSV at indicated time points. PAMs with a UBE2L6 knockdown promoted the mRNA expression of *ISG15* at 3 and 6 hpi. The expression of *ISG56* was slightly induced as early as 3 hpi and significantly up-regulated to 12 hpi in UBE2L6-silenced PAMs (Figure 7C).

Compared with the negative control, knockdown of UBE2L6 also accelerated type I interferon (*IFN-α* and *IFN-β*) mRNA expression during PRRSV infection (Figure 7C). These results illustrated that UBE2L6 inhibits the transcription of type I interferon and interferon-stimulated genes, thus facilitating PRRSV replication.

### UBE2L6 stabilised the viral NSP5 protein

**Figure 8:**
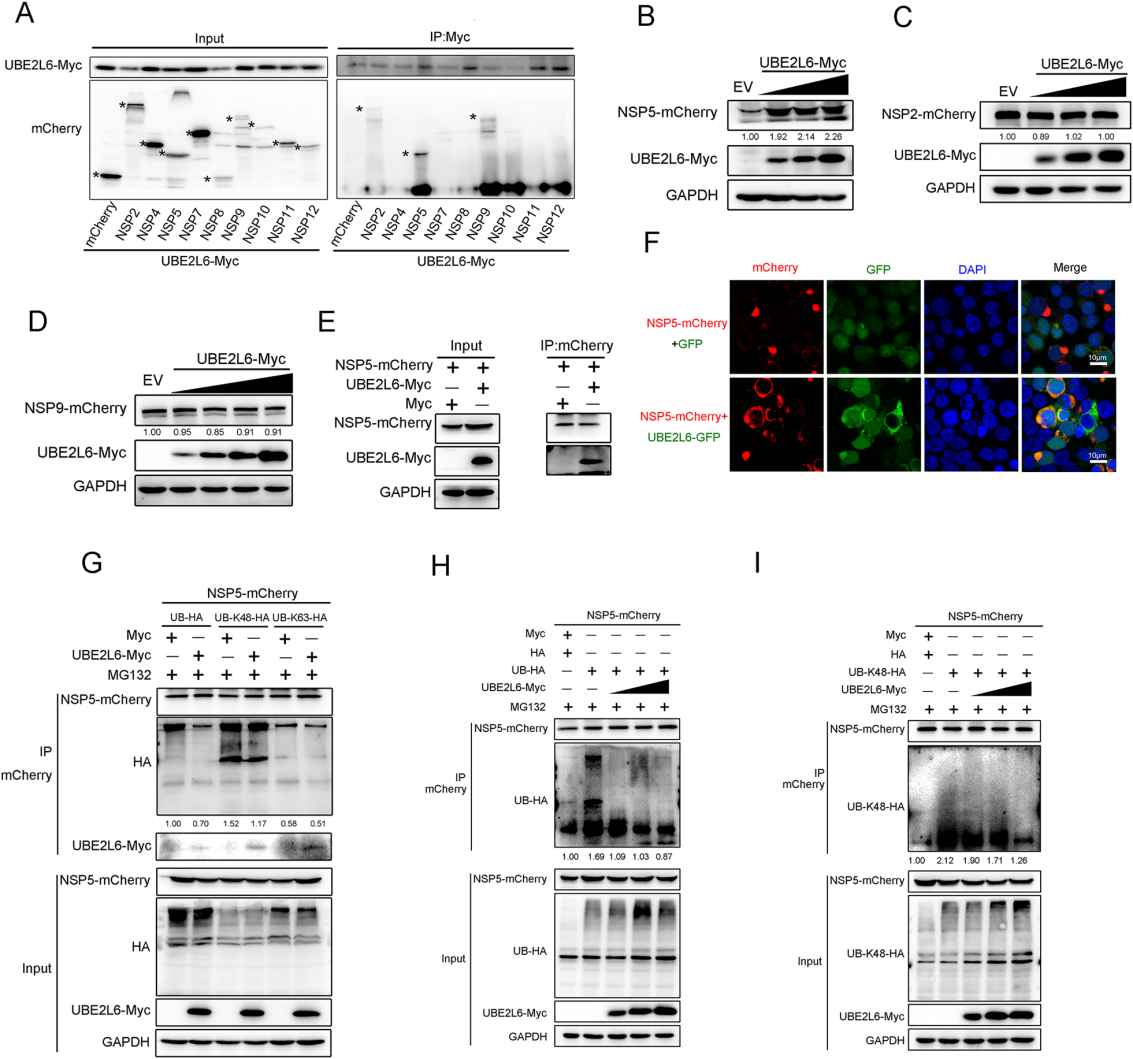
**UBE2L6 interacted with PRRSV NSP5 and stabilised NSP5 protein expression.** **A** HEK293T cells were co-transfected with Myc-tagged *UBE2L6* plasmids and different viral protein plasmids, respectively. Cell lysates were immunoprecipitated with a Myc antibody. Immunoblots of Myc-tagged UBE2L6 and mCherry-tagged viral proteins were shown. Asterisks mark the expressed mCherry-fusion proteins of viral proteins. **B**–**D** HEK293T cells were transfected with control plasmids and different concentrations of Myc-tagged *UBE2L6* plasmids, together with mCherry-tagged PRRSV *NSP5* plasmids (**B**), PRRSV *NSP2* plasmids (**C**), or PRRSV *NSP9* plasmids (**D**), respectively. Viral protein exogenous expression was detected using an mCherry antibody, as shown by western blot. GAPDH was shown as an internal control. **E** mCherry-tagged PRRSV *NSP5* plasmids and Myc-tagged *UBE2L6* plasmids were co-transfected into HEK293T cells for 24 h. Co-IP assay was performed using an mCherry antibody, and immunoblots with Myc and mCherry antibodies were shown. **F** mCherry-tagged PRRSV *NSP5* and GFP-tagged *UBE2L6* were co-transfected into HEK293T cells for 24 h. Immunofluorescence analysis of the co-localisation between PRRSV NSP5 (red) and UBE2L6 (green) is shown. (Bar, 10 µm). **G** HEK293T cells were co-transfected with mCherry-tagged *Nsp5* plasmids and Myc-tagged *UBE2L6* plasmids, additionally with HA-tagged Ub, HA-tagged Ub-K48, or HA-tagged Ub-K63 plasmids for 24 h, respectively. Cells were treated with MG132 (10 mM) for another 6 h. Cell lysates were immunoprecipitated with an mCherry antibody, and immunoblots of HA, Myc, and mCherry are shown using the indicated antibodies. **H**, **I** HEK293T cells were transfected with mCherry-tagged *NSP5*, HA-tagged Ub (**H**) or HA-tagged Ub-K48 (**I**), and different concentrations of Myc-tagged *UBE2L6* for 24 h. Cells were treated with MG132 (10 mM) for another 6 h. Cells were lysed for co-IP using an antibody against mCherry, and immunoprecipitates were analysed by western blotting with HA and mCherry antibodies. The relative band density was normalised to the loading control GAPDH and then compared to the corresponding control.

UBE2L6 suppresses the innate immune response by degrading the expression of RIG-I and MDA5 and by inhibiting ISGylation. Interestingly, the process by which UBE2L6 inhibits innate immunity requires the participation of PRRSV. To identify which viral proteins might be involved in UBE2L6-regulated signalling, we screened for the viral protein potentially associated with this pathway. HEK293T cells were co-transfected with mCherry-tagged PRRSV non-structural protein plasmids and Myc-tagged *UBE2L6* plasmids. Cell extracts were then subjected to immunoprecipitation and immunoblotting. Co-IP assays revealed that UBE2L6 interacted with viral NSP2, NSP5, and NSP9, as shown by immunoprecipitation using Myc antibody-conjugated beads (Figure 8A). Next, HEK293T cells were co-transfected with different concentrations of Myc-tagged *UBE2L6* and mCherry-tagged *NSP2, NSP5*, or *NSP9* plasmids. As shown in Figure 8B, increasing expression of UBE2L6 led to a stable and consistent increase in NSP5 protein expression levels. However, no visible changes were observed in the expression of NSP2 and NSP9, even when UBE2L6 was overexpressed (Figures 8C, D).

In addition, empty vector or *UBE2L6*-expressing plasmids were co-transfected with mCherry-tagged *NSP5*, and co-IP assays were conducted to confirm the interaction between NSP5 and UBE2L6. As expected, the mCherry antibody successfully immunoprecipitated Myc-tagged UBE2L6 (Figure 8E). Exogenous GFP-plasmid or *UBE2L6*-GFP-plasmid was co-transfected into HEK293T cells with mCherry-tagged NSP5. The NSP5 was distinctly co-localised with UBE2L6, as shown by fluorescence analysis (Figure 8F). These data indicated that UBE2L6 interacts with NSP5. To further demonstrate how UBE2L6 affects NSP5 expression stability, we sought to detect NSP5’s ubiquitination level in the presence of UBE2L6 overexpression. HEK293T cells were co-transfected with mCherry-tagged *NSP5* and Myc-tagged *UBE2L6* plasmid, along with either Ub-HA, Ub-48K-HA, and Ub-63K-HA plasmids. We observed that UBE2L6 overexpression led to a noticeable decrease in the total ubiquitination level of NSP5. Specifically, K48-linked ubiquitination was markedly reduced in exogenous UBE2L6-expressing cells, while K63-linked ubiquitination remained almost unchanged regardless of whether UBE2L6 existed (Figure 8G). The results suggest that UBE2L6 inhibits the degradation of NSP5 by lowering its K48-linked ubiquitination and thus plays a role in stabilising NSP5 protein.

To further validate this mechanism, HEK293T cells were transfected with mCherry-tagged *NSP5*, Ub-HA, and varying concentrations of *UBE2L6* plasmids. Immunoprecipitation using mCherry antibody showed that the total ubiquitination level of NSP5 decreased in a dose-dependent manner as UBE2L6 expression increased (Figure 8H). Similarly, the K48-linked ubiquitination level in NSP5 was also reduced upon UBE2L6 overexpression in a dose-dependent manner (Figure 8I). Taken together, these results demonstrate that UBE2L6 stabilises the viral NSP5 protein by impairing K48-linked ubiquitination.

### PRRSV NSP5 combined with UBE2L6 to further promote the degradation of RIG-I and MDA5

**Figure 9:**
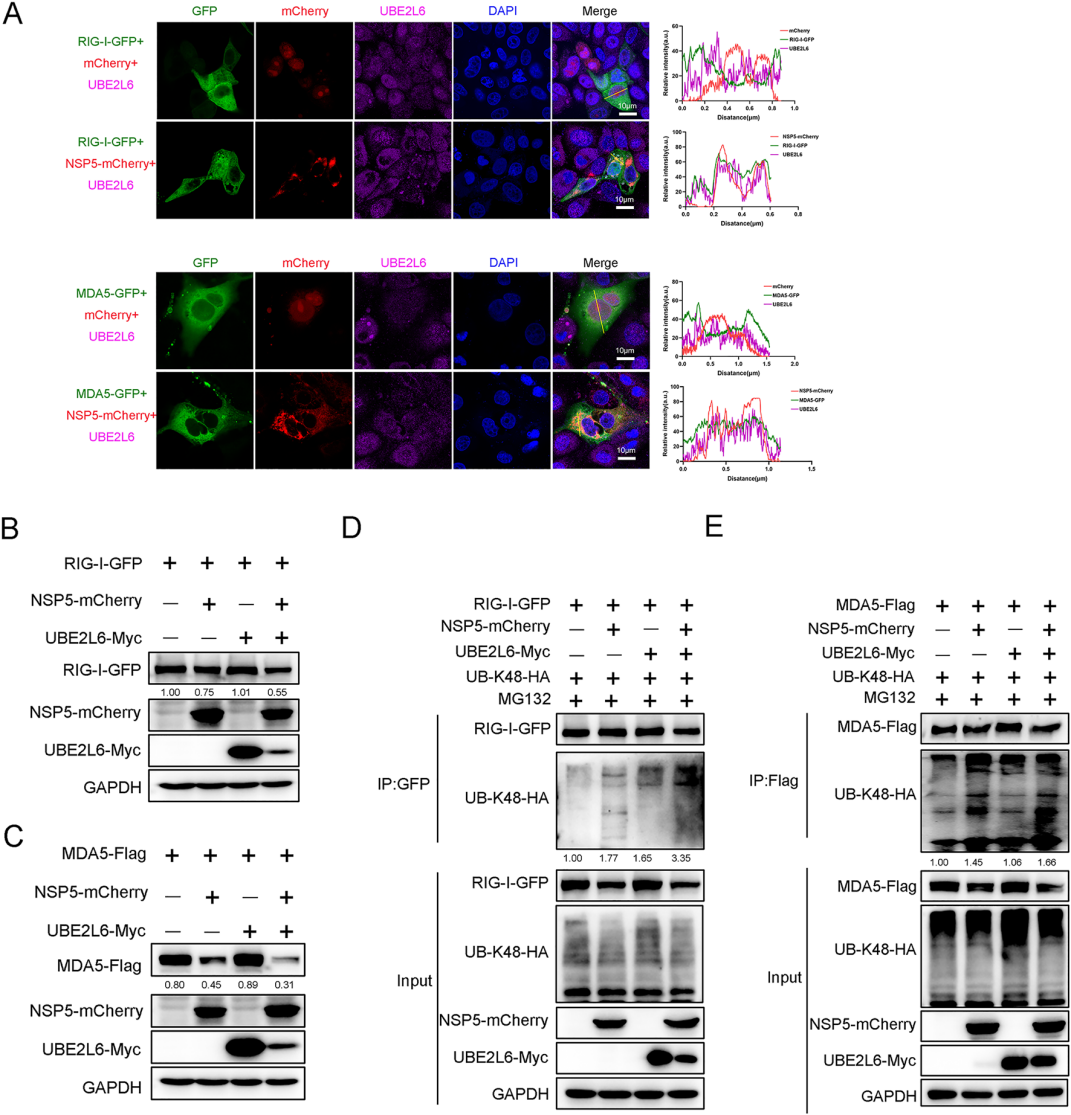
**PRRSV NSP5 facilitated UBE2L6-mediated RLRs degradation via the ubiquitin–proteasome pathway.** **A** PK-15 cells were transfected with GFP-tagged *RIG-I* plasmids and mCherry-tagged *NSP5* plasmids for 24 h. Endogenous UBE2L6 was detected by using a UBE2L6 antibody and a corresponding fluorescent secondary antibody. Immunofluorescence analysis of the colocalisation among RIG-I (green), NSP5 (red), UBE2L6 (purple), and DAPI (blue) was performed. Moreover, PK-15 cells were also transfected with GFP-tagged *MDA5* plasmids and mCherry-tagged *NSP5* plasmids for 24 h. Endogenous UBE2L6 was detected by using a UBE2L6 antibody. The location of MDA5 (green), NSP5 (red), UBE2L6 (purple), and DAPI (blue) in the cells was shown by immunofluorescence. Scale bar, 10 μm. Intracellular colocalisation of these proteins was analysed by ImageJ software (right column). **B** HEK293T cells were transfected or co-transfected with GFP-tagged *RIG-I* plasmids, control plasmids, mCherry-tagged *NSP5* plasmids, and Myc-tagged *UBE2L6* plasmids, respectively. Western blot was used to detect the expression of RIG-I, NSP5, and UBE2L6 using indicated antibodies. GAPDH was shown as an internal control. **C** HEK293T cells were transfected or co-transfected with Flag-tagged *MDA5* plasmids, control plasmids, mCherry-tagged *NSP5* plasmids, and Myc-tagged *UBE2L6* plasmids, respectively. Western blot was used to detect the expression of MDA5, NSP5, and UBE2L6 using indicated antibodies. GAPDH was shown as an internal control. **D** HEK293T cells were all transfected with GFP-tagged *RIG-I* and HA-tagged Ub-K48 plasmids, additionally co-transfected with control plasmids, mCherry-tagged *NSP5*, or Myc-tagged *UBE2L6* plasmids, respectively. IP assays with anti-GFP antibody were performed to determine the K48-linked ubiquitination level of RIG-I. Immunoblots of RIG-I, K48 ubiquitination, UBE2L6, and NSP5 with indicated tag antibodies are shown. GAPDH was shown as an internal control. **E** HEK293T cells were co-transfected with Flag-tagged *MDA5* and HA-tagged Ub-K48 plasmids, accompanied by control plasmids, mCherry-tagged *NSP5*, or Myc-tagged *UBE2L6*, respectively. Cell lysates were immunoprecipitated with a Flag antibody, and immunoblots are shown to detect the K48-linked ubiquitination in MDA5 protein in the presence of UBE2L6 and NSP5. Flag, Myc, HA, and mCherry tag antibodies were used for western blot. GAPDH was shown as an internal control. The relative band density was normalised to the loading control GAPDH and then compared to the corresponding control.

NSP5 was recently reported to contribute to PRRSV-induced immunosuppression [11]. In this study, we observed that UBE2L6 down-regulated the host innate immune response and stabilised the viral NSP5 with which it physically interacted. Accordingly, we hypothesised that NSP5 and UBE2L6 could synergistically degrade RIG-I or MDA5, thereby inhibiting the innate immune response. To test this, GFP-tagged *RIG-I* or GFP-tagged *MDA5* plasmid was co-transfected with mCherry-tagged *NSP5* plasmid into PK-15 cells. A UBE2L6 antibody was used to detect endogenous UBE2L6 protein, and co-localisation was shown by immunofluorescence analysis. As shown in Figure 9A, there was a clear co-localisation among exogenous NSP5, RIG-I, and endogenous UBE2L6. Additionally, immunofluorescence assays showed that NSP5, MDA5 and UBE2L6 could co-localise with each other when compared to the control group. These data suggest that NSP5 could potentially participate in UBE2L6-mediated RIG-I and MDA5 degradation.

Subsequently, HEK293T cells were transfected either individually or in combination with GFP-tagged *RIG-I,* mCherry-tagged *NSP5*, or Myc-tagged *UBE2L6* plasmids. As expected, compared with GFP-tagged *RIG-I* plasmid transfected alone, UBE2L6 could not reduce the expression of RIG-I in the absence of NSP5. However, the expression of RIG-I was slightly reduced in NSP5-overexpressing cells and declined further when NSP5 and UBE2L6 were co-expressed (Figure 9B). Similarly, there was no noticeable change in the expression of MDA5 when cells were co-transfected with *MDA5* and *UBE2L6*. A pronounced reduction of MDA5 expression occurred once NSP5 and UBE2L6 coexisted in the cells (Figure 9C). These findings indicate that UBE2L6 requires NSP5 to mediate the degradation of RIG-I and MDA5 and that NSP5 promotes UBE2L6-induced RIG-I and MDA5 degradation.

After demonstrating that UBE2L6 suppresses RIG-I and MDA5 expression through K48-linked ubiquitination during PRRSV infection, we next investigated whether PRRSV NSP5 utilised UBE2L6 to facilitate ubiquitination degradation of RIG-I and MDA5. HEK293T cells were transfected with GFP-tagged *RIG-I*, Ub-48K-HA, mCherry-tagged *NSP5*, and Myc-tagged *UBE2L6*, alone or in combination. GFP antibody was used to immunoprecipitate and detect the K48-linked ubiquitination level in RIG-I. As shown in Figure 9D, compared with the control group, the K48-linked ubiquitination level of RIG-I was significantly elevated in the NSP5-expressing protein cells and further increased when both NSP5 and UBE2L6 were co-expressed. Simultaneously, Flag-tagged *MDA5* plasmids were used to identify the K48-linked ubiquitination level in MDA5. PRRSV NSP5 significantly promoted the K48-linked ubiquitination level of MDA5 in UBE2L6-expressing cells compared with other control groups (Figure 9E).

These results demonstrate that PRRSV NSP5 is essential for UBE2L6-mediated degradation of RIG-I and MDA5. PRRSV NSP5 appears to recruit UBE2L6 binding to RIG-I and MDA5, ultimately promoting their degradation via increasing the K48-linked ubiquitination level. This cooperative mechanism suppresses the innate immune response and facilitates PRRSV replication.

## Discussion

PRRSV is a significant pathogen affecting the global swine industry, causing substantial economic losses [26]. One of the key challenges in controlling PRRSV lies in its capacity for immunosuppression. PRRSV suppresses the host innate and adaptive immune responses, resulting in low levels of neutralising antibodies and effective immune escape. As such, this mechanism deserves further investigation [27].

**Figure 10:**
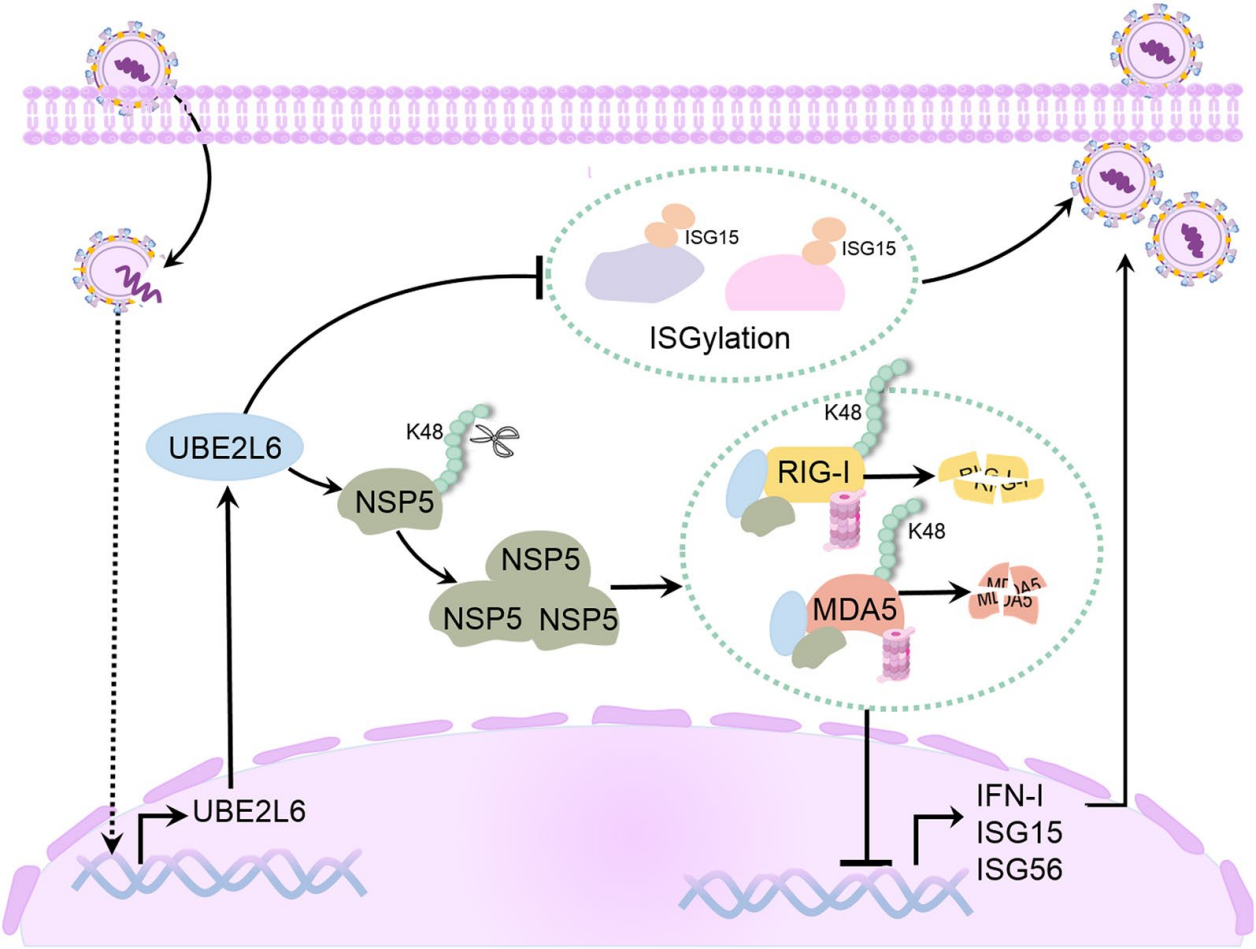
**UBE2L6 promoted PRRSV replication pathways.** Upon PRRSV infection, the mRNA and protein expression of UBE2L6 were up-regulated. UBE2L6 overexpression lowered host ISGylation level during PRRSV infection. Meanwhile, increased UBE2L6 impaired K48-linked ubiquitination in PRRSV NSP5 and stabilised NSP5 protein. PRRSV NSP5 utilised UBE2L6 to further promote the degradation of RIG/MDA5 via K48-linked ubiquitination pathway, thus restraining the expression of type I interferons and ISGs and facilitating PRRSV replication eventually.

In this study, we provide new insights into the mechanism of how PRRSV inhibits the type I interferon response, as summarised in Figure 10. We found that PRRSV infection promoted the mRNA and protein expression of *UBE2L6*. This overexpression facilitates PRRSV replication, whereas silencing of *UBE2L6* inhibits it. Mechanistically, increased levels of UBE2L6 interact with PRRSV NSP5 and stabilise the viral NSP5 protein by impairing its K48-linked ubiquitination. In turn, this stabilised PRRSV NSP5 synergistically with UBE2L6 to degrade the expression of RIG-I and MDA5 by facilitating their K48-linked ubiquitination, ultimately suppressing the expression of type I interferon and promoting PRRSV replication. UBE2L6 functions as an E2 ubiquitin/ISG15-conjugating enzyme and is known to influence both proteasomal degradation and ISGylation. In this study, we identified that UBE2L6 may play multiple roles in regulating innate immunity, particularly during PRRSV infection.

We identified that under normal conditions, *UBE2L6* overexpression did not affect the expression of RIG-I and MDA5 but increased host ISGylation (Figure 5). Interestingly, once PRRSV infection, RIG-I and MDA5 expression were markedly reduced, and ISGylation was also inhibited in UBE2L6 overexpressed cells (Figure 4A). These results suggested that PRRSV was required for UBEL6-mediated inhibition of innate immune response, and viral proteins may be involved in this process. The innate immune system represents the host’s first line of defence against pathogen infection. PRRs play a critical role in detecting viral RNA or DNA molecules from pathogenic microorganisms. Among these, RIG-I-like receptors (RLRs), including RIG-I, MDA5, and LGP2, recognise viral nucleic acids and form a complex with MAVS for downstream signalling molecules. This process leads to nuclear translocation of IRF3, IRF7 or NF-ĸB and the subsequent induction of type I interferon and inflammatory cytokines [28].

PRRSV is an immunosuppressive pathogen that inhibits host innate immunity through multiple mechanisms, including interference with the RIG-I signalling pathway [29]. However, the specific function of PRRSV NSP5 and whether it antagonises host antiviral innate immunity has been rarely addressed. Previous studies have shown that PRRSV NSP5 is mainly involved in the autophagy process [30], including degradation of STAT3 and inhibition of JAK/STAT3 signalling [31]. Furthermore, PRRSV NSP5 also degrades the RLR signalling pathway via FAM134B-mediated autophagy [11]. In contrast, our findings demonstrate that PRRSV NSP5 utilised ubiquitin conjugates instead of autophagy to degrade the RLRs signalling pathway. We observed that both PRRSV infection and PRRSV NSP5 transfection inhibited the expression of RIG-I and MDA5, which is consistent with previous studies. However, when *UBE2L6* was overexpressed, PRRSV or PRRSV NSP5 induced higher RIG-I and MDA5 degradation than PRRSV infection or NSP5 plasmid transfection alone (Figures 4 and 9).

Moreover, the K48-linked ubiquitination levels of RIG-I and MDA5 were visibly elevated in the presence of *UBE2L6* overexpression during PRRSV infection (Figures 6D, E). In contrast, *UBE2L6* overexpression did not affect RIG-I and MDA5 expression in the absence of viral infection or NSP5 plasmid transfection, even when UBE2L6 was overexpressed (Figure 5). These data suggested that UBE2l6 had little effect on the expression of RIG-I and MDA5 under normal conditions. Upon PRRSV infection, NSP5 serves as a scaffold and promotes UBE2L6 binding to RIG-I and MDA5, thus accelerating their ubiquitination degradation. PRRSV NSP5 itself does not function as a ubiquitination-associated enzyme, but it bridges the interaction between UBE2L6 and RLRs. Our findings provide new insights into the immunosuppression caused by PRRSV NSP5 during infection. Whether PRRSV NSP5 also acts as a scaffold for other E2 or E3 ubiquitin ligases or other viral proteins that inhibit innate immunity in this way needs further investigation.

According to our data, PRRSV NSP5 utilises UBE2L6 to promote the degradation of RIG-I and MDA5, thereby inhibiting the host innate immune response. In this process, PRRSV and UBE2L6 appear to reinforce and complement each other. On one hand, PRRSV infection promoted both the mRNA and protein expression of *UBE2L6* (Figure 1). On the other hand, UBE2L6 unexpectedly impairs PRRSV NSP5 K48-linked ubiquitination and stabilises viral NSP5 protein (Figure 8). During SVA infection, UBE2L6 has been shown to interact with the viral 3D protein and increase its total K48-linked and K63-linked ubiquitination levels to improve its stability [23].

In contrast, our study revealed that the total ubiquitination level and K48 chains in PRRSV NSP5 are reduced during PRRSV infection. Although UBE2L6, as an E2-conjugating enzyme, might be expected to facilitate the ubiquitination of NSP5, as it does with RIG-I and MDA5. However, our results indicate the opposite: UBE2L6 inhibits the ubiquitination levels of NSP5. This discrepancy implies that other viral or host proteins may be involved in counteracting this ubiquitination process. Further investigation is needed to clarify these interactions. Moreover, we identified that other viral proteins, NSP2 and NSP9, could also interact with UBE2L6. However, UBE2L6 had no effects on their expression (Figures 8C, D). Both NSP2 and NSP9 are essential viral proteins responsible for PRRSV replication, and NSP2, in particular, contains a deubiquitinating domain [32]. We speculate that these proteins may hijack UBE2L6 to perform their deubiquitination and promote viral replication.

The ubiquitin-like protein ISG15 is induced by type I interferons and subsequently participates in an enzymatic cascade to mediate ISGylation. ISGylation has been shown to play an important role in the antiviral innate immune responses [33, 34]. For example, ISGylation of STAT1 preserves its phosphorylation and activation, whereas suppression of STAT1 ISGylation promotes hepatitis C virus (HCV) pathogenesis [35]. Similarly, ISGylation of IRF3 maintains its activation by attenuating interaction with PIN1, thereby inhibiting SeV replication [36]. ISGylation of PKR, JAK1, ERK1, IFIT1, and MxA also facilitates the production of type I interferons and ISGs [12].

In addition to host proteins, ISGylation of viral proteins also inhibits viral evasion and replication. For instance, the IAV NS1 protein functions as a substrate for ISG15, and the ISGylation of this protein blocks its nuclear localisation and inhibits virus replication [37]. Coxsackievirus B3 (CVB3) 2A^pro is a potential target for ISGylation, which inhibits its protease activity, restores host protein translation and inhibits CVB3 replication [17]. ISGylated infectious bronchitis virus (IBV) NPs and ISGylated human papillomavirus (HPV)-16 capsid protein also block viral RNA synthesis and the release of viral particles, respectively [12, 16, 38].

In our study, we observed an increase in total ISGylation levels in cells during PRRSV infection (Figure 1A). The elevated ISGylation level showed strong anti-PRRSV activity. However, when ISGylation was disrupted using a conjugation-defective ISG15 mutant, PRRSV replication was restored (Figure 4D). Similar to other viruses, the extent of ISGylation plays a crucial role in regulating PRRSV replication. We speculate that ISGylation promotes the host innate immune response during PRRSV infection, or viral proteins are ISGylated, which disrupts their assembly or function, or interferes with their interaction with host pathways. These outcomes ultimately result in the inhibition of viral replication. The findings and hypothesis of ISGylation on PRRSV merit further investigation.

UBE2L6, as an ISG15-conjugating enzyme, is crucial for the enzymatic cascade of ISG15 conjugation. As expected, in the absence of PRRSV infection, UBE2L6 overexpression increased the ISGylation level in cells. However, interestingly, once cells were infected with PRRSV, the level of ISGylation was decreased even when UBE2L6 was overexpressed. This suggests that PRRSV or viral-encoded proteins actively play an essential role in this process. We propose two possible mechanisms for this effect. First, PRRSV or its viral proteins may interfere with the interaction between UBE2L6 and its substrate, or E3 ISGylation ligase, thus antagonising UBE2L6-mediated ISGylation for its replication. Alternatively, PRRSV may hijack UBE2L6 to prioritise its role in ubiquitination, particularly of RLRs, so that it diminishes ISGylation signalling cascades.

In conclusion, we described the mechanism by which PRRSV NSP5 exploits UBE2L6 to regulate the host innate immune response. We found that specifically, PRRSV infection promoted *UBE2L6* expression, and in turn, UBE2L6 overexpression inhibited ISGylation level during PRRSV infection. UBE2L6 also interacts with PRRSV NSP5 and stabilises viral NSP5 expression. Stable PRRSV NSP5, in combination with UBE2L6, significantly promotes RIG-I and MDA5 degradation through K48-linked ubiquitination, thereby reducing the expression of type I interferons and ISGs while facilitating PRRSV replication. These findings provide new insights into how PRRSV inhibits the host innate immune response, offering a potential basis for preventing and controlling PRRSV.

## Data Availability

All data generated during this study are included in this published article. The raw data generated during the current study are available from the corresponding author upon reasonable request.
